# Direct/Conjugated Hyperbilirubinemia as an Uncommon Presentation of Acute Alcoholic Hepatitis: A Case Report

**DOI:** 10.7759/cureus.37548

**Published:** 2023-04-13

**Authors:** Mohammad Abu-Abaa, Salman Kananeh, Aliaa Mousa, Omar Jumaah

**Affiliations:** 1 Internal Medicine, Capital Health Regional Medical Center, Trenton, USA

**Keywords:** alcohol misuse, acute alcoholic hepatitis, alcohol-related liver disease, jaundice cholestatic, direct hyperbilirubinemia

## Abstract

Alcoholic liver disease (ALD) is a common pathology in clinical practice and is clinically diverse. Acute alcoholic hepatitis is an acute inflammation of the liver with or without underlying cholestasis and steatosis. In this case, we are presenting a 36-year-old male with a past medical history of alcohol use disorder who presented with two weeks of right upper quadrant abdominal pain and jaundice. However, direct/conjugated hyperbilirubinemia with relatively low aminotransferases in labs prompted investigation for obstructive and autoimmune hepatic pathologies. Unrevealing investigations prompted consideration of acute alcoholic hepatitis with cholestasis and a course of oral corticosteroids that gradually improved the patient’s clinical symptoms and liver function test. This case helps to remind clinicians that although ALD is usually associated with indirect/unconjugated hyperbilirubinemia and elevated aminotransferases, presentation of ALD with mainly direct/conjugated hyperbilirubinemia with relatively low aminotransferases is a possibility. Although imaging tests should be pursued to rule out obstructive etiologies, invasive tests and liver biopsies are not indicated in typical clinical settings.

## Introduction

Alcoholic liver disease (ALD) includes a spectrum of dysplastic changes of hepatocytes and ranges from alcoholic fatty liver disease, alcoholic steatohepatitis and/or alcoholic hepatitis, to liver cirrhosis. Patients can be initially asymptomatic but eventually present with stigmata of liver disease including jaundice [1]. Cholestasis can be seen in all stages of ALD. Cholestasis refers to bile acid retention in hepatocytes forming bile acid thrombi on histological examination [2]. However, hyperbilirubinemia in ALD is usually of unconjugated/indirect type, and presentation of ALD with isolated conjugated hyperbilirubinemia and jaundice with otherwise relatively normal liver function test is extremely rare and naturally prompts extensive workup to rule out obstructive etiologies [1].

## Case presentation

A 36-year-old male patient presented to the emergency department (ED) with right upper abdominal quadrant pain and scleral jaundice for two to three weeks prior to presentation. He described the pain as dull, intermittent, mild to moderate, non-related to food, non-radiating, and associated with nausea with no vomiting or fever. He also admitted to dark discoloration of the urine. He denied a history of illicit drug use and unprotected sexual contact. His past medical history was remarkable only for alcohol use disorder, and he reported drinking 10 cans of beer and one bottle of Vodka daily for eight years. In the ED, vital signs included a temperature of 37.2°C, heart rate of 96 beats per minute, respiratory rate of 18 cycles per minute, blood pressure of 150/70 mmHg, and SpO_2_ of 99% on room air. On a physical exam, he had scleral jaundice and right upper abdominal quadrant superficial tenderness with a negative Murphy sign. Evidence of hepatomegaly was also appreciated clinically. Otherwise, the physical exam was unremarkable. Basic labs showed elevated total bilirubin of 7.8 mg/dL mainly of a direct type of 6.1 mg/dL, elevated alkaline phosphatase of 216 U/L, elevated aspartate transaminase (AST) of 80 U/L with normal alanine transaminase (ALT).

**Figure 1:**
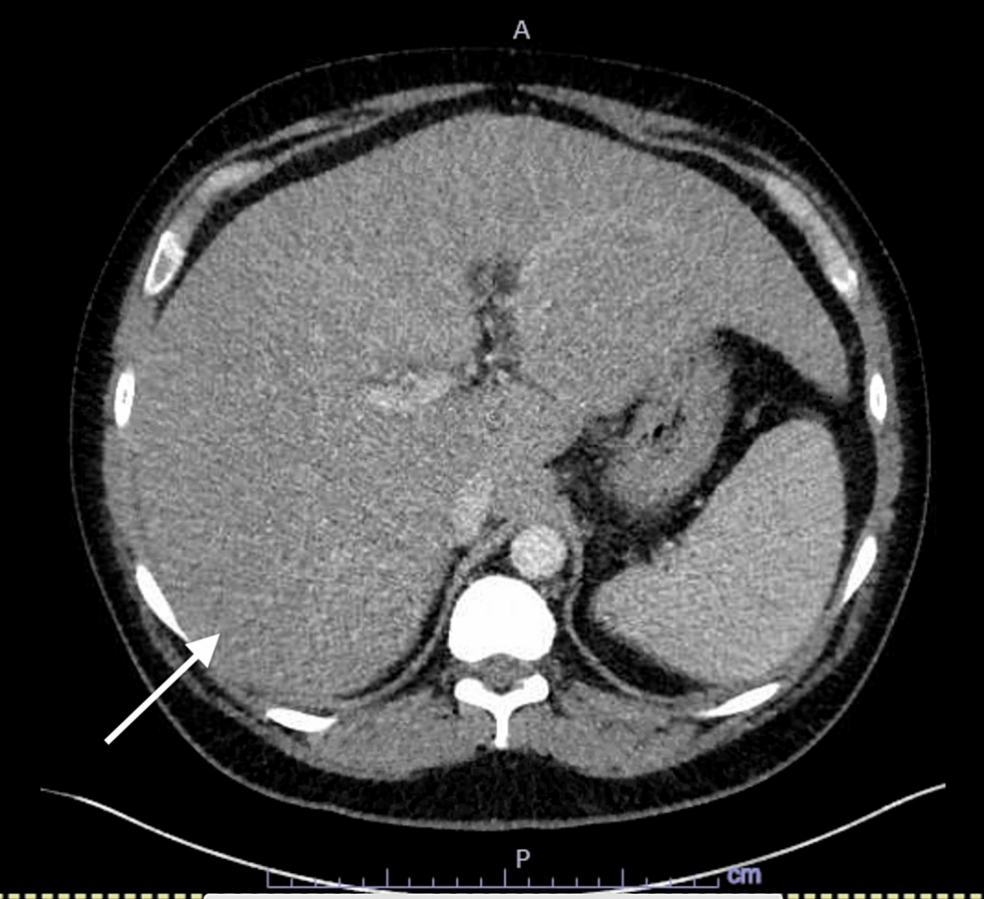
Cross-sectional computed tomography scan of the abdomen Cross-sectional abdominal computed tomography (CT) scan showing evidence of hepatomegaly (arrow).

**Figure 2:**
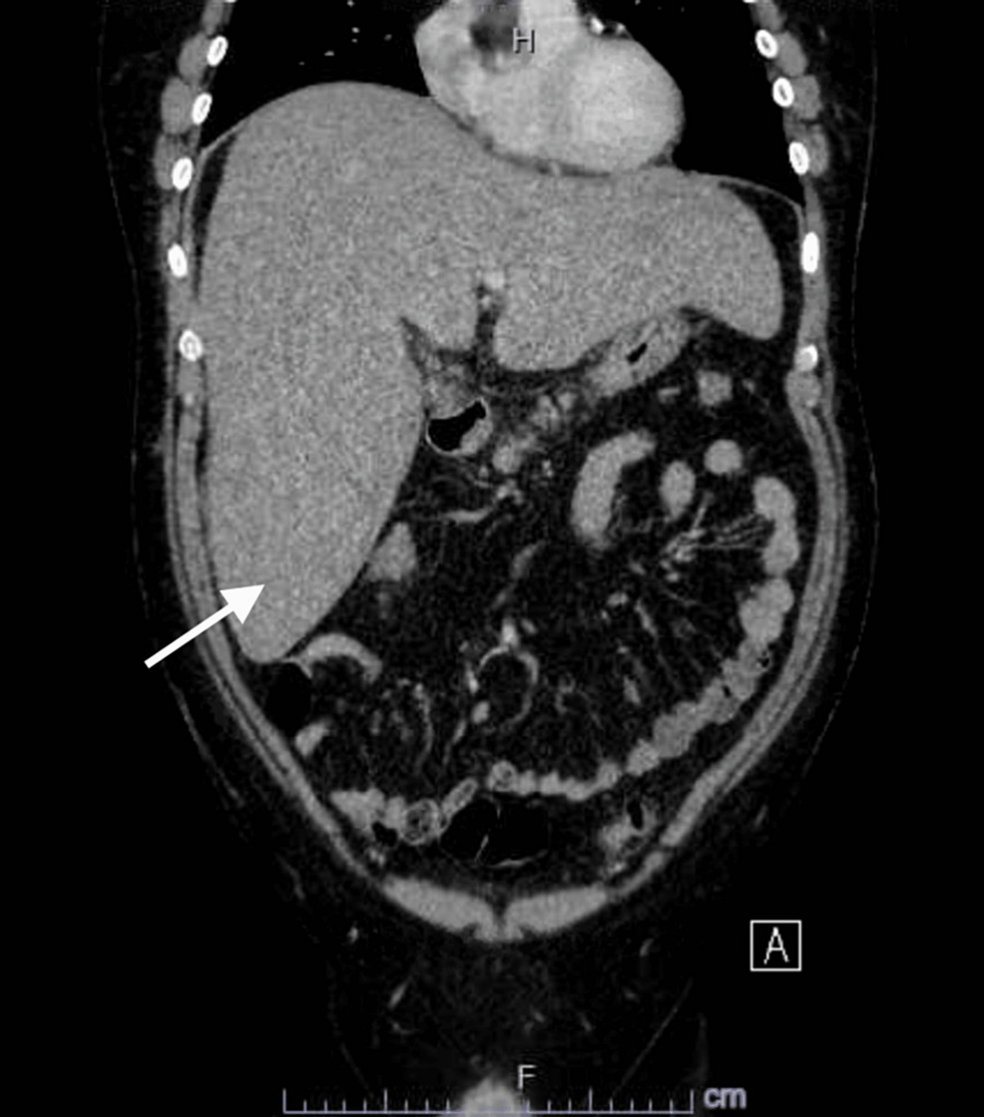
Coronal CT scan of the abdomen Coronal computed tomography (CT) scan of the abdomen showing evidence of hepatomegaly (arrow).

**Figure 3:**
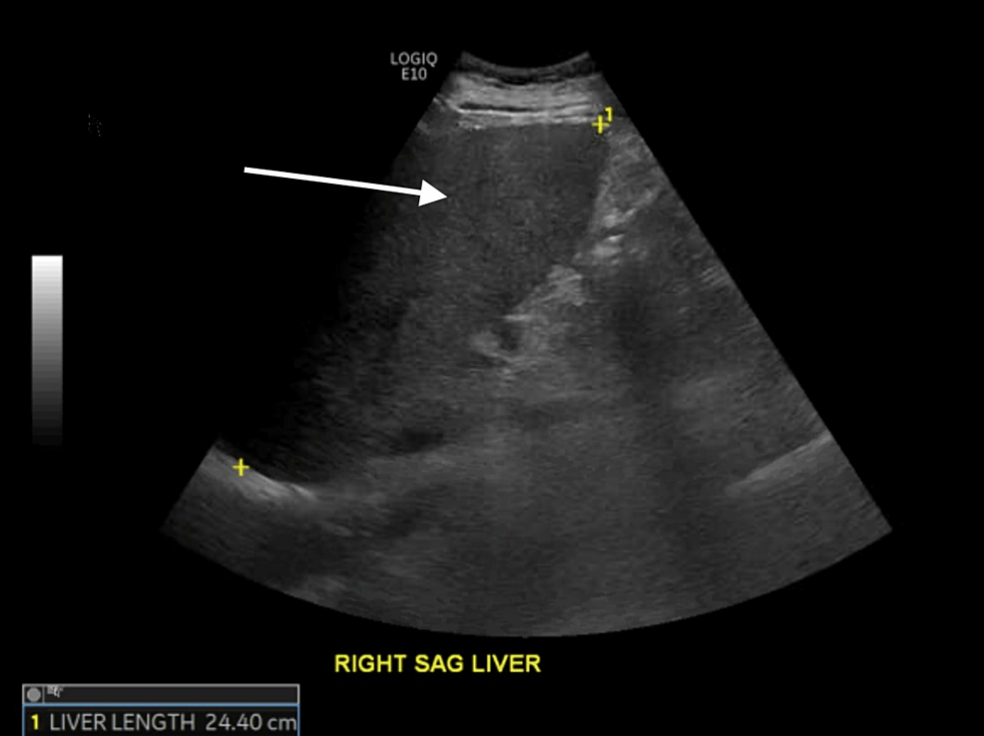
Abdominal ultrasound Abdominal ultrasound showing reduced hepatic echogenicity with hepatic enlargement suggestive of hepatic fatty infiltration (arrow).

Computed tomography (CT) scan of the abdomen and pelvis showed hepatomegaly with low attenuation suggestive of hepatic fatty infiltration (Figures 1, 2). Liver ultrasound showed heterogeneously increased echogenicity of the liver suggestive of hepatic steatosis with enlarged liver around 24.4 cm in the midclavicular line with no hypoechoic masses or intrahepatic biliary dilation and normal portal flow. It also showed a normal gallbladder with a normal common bile duct of 0.4 cm proximally and distally (Figure 3).

While being hospitalized, it was noted that total bilirubin continued to trend higher at 9.8 mg/dL mainly of direct bilirubin of 8.3 mg/dL on the second day of admission (see Table 1 for lab trends). Gamma-glutamyl transpeptidase (GGT) was elevated at 677 international unit/L (reference less than 45). The hepatitis panel was negative for hepatitis B, C, and A. Autoimmune profile including anti-smooth muscle, anti-mitochondrial M2, and liver-kidney microsomal antibodies were negative. The patient was diagnosed with alcoholic hepatitis with elevated Maddrey’s discriminant function of 32. He was started on oral prednisone with gradual improvement of total serum bilirubin down to 7.1 mg/dL. The patient improved clinically allowing for discharge.

**Table 1:** Lab trends This shows a tabular format of lab trends throughout the course of hospitalization. WBC: White blood cell; MCV: Mean corpuscular volume; PT: Prothrombin time; INR: International normalized ratio; AST: Aspartate transaminase; ALT: Alanine transaminase.

Variables	On Admission	Day 2	Day 3	Day 4	Day 7	References
WBC	10.88	11.88	12.26	-	-	4000-10,0000 cells/mcL
Hemoglobin	13	12.7	13	-	-	13.7-17.5 g/dL
MCV	97.2	96.3	95.9	-	-	79-95 fL
Platelet	205	198	212	-	-	150-400 cells/mcL
PT	16.4	16.7	16.7	17.4	16	12.4-14.8 seconds
INR	1.3	1.3	1.3	1.4	1.3	0.8-1.1
Sodium	132	131	131	129	133	137-145 mmol/L
Creatinine	0.5	0.5	0.6	0.5	0.5	0.6-1.25 mg/dL
Total bilirubin	7.8	9.8	12	14.8	7.1	0.2-1.3 mg/dL
Direct bilirubin	6.1	8.3	10.2	10.8	4.6	0-0.4 mg/dL
Alkaline phosphatase	260	230	220	238	212	38-126 U/L
AST	80	75	66	61	54	17-59 U/L
ALT	18	18	18	22	21	0-49 U/L

## Discussion

Two-thirds of US adults drink alcohol, and 7.2% have a diagnosis of alcohol use disorder. According to the National Institute on Alcohol Abuse and Alcoholism (NIAAA), the diagnosis of alcoholic hepatitis is established with the following criteria: onset of jaundice within 60 days of heavy alcohol use of more than 50 g/days for at least six months, elevated serum bilirubin more than 3 g/dL, elevated AST between 50 and 400 U/L, and AST/ALT ratio more than 2 with no other identifiable etiologies of hepatitis [3].

Alcoholic hepatitis is characterized by the acute onset of symptomatic hepatitis with symptoms of acute right upper quadrant abdominal pain, fever, fatigue, tender hepatomegaly, and jaundice [4]. Hyperbilirubinemia in ALD is usually of unconjugated type, and conjugated hyperbilirubinemia is rare in ALD. In addition, the presentation of ALD with isolated hyperbilirubinemia and relatively normal aminotransferases is extremely rare with only a few reported cases [5-7]. A case series of intrahepatic cholestasis showed excellent recovery as compared to life-threatening presentations of ALD [8].

The exact mechanism of alcohol-induced cholestasis is largely unknown. However, possible mechanisms have been suggested by animal studies. Normally, the transport of bile acids from hepatocytes to biliary canaliculi is achieved by both bile acid-dependent and bile acid-independent mechanisms. The latter include chemical- and electrical-driven transport facilitated by sodium-potassium ATPase pump. This can be inhibited by alcohol. In addition, the transcytosis of bile acids in hepatocytes can also be inhibited by alcohol [9-11].

In addition to the rarity of this presentation of ALD, this case serves to remind clinicians that liver biopsy is not indicated in this presentation as histological changes can be indistinguishable between alcoholic and non-alcoholic liver disease [12]. Unlike alcoholic steatohepatitis which is a histological diagnosis, the diagnosis of alcoholic hepatitis is mainly clinical. In addition to history, lab clues for ALD include AST/ALT ratio of more than 2 and elevated GGT [13]. Generally, diagnosis of ALD can be established based on clinical and laboratory features in those with a long-standing history of heavy alcohol use. It is reasonable to pursue abdominal imaging especially abdominal ultrasound to rule out obstructive causes. However, liver biopsy is rarely required [3].

## Conclusions

This case is demonstrating an atypical presentation of ALD with only direct/conjugated hyperbilirubinemia and relatively low AST/ALT. Awareness of this possible presentation helps to solve a diagnostic dilemma and spare the patient from invasive testing. In a typical clinical setting with a suggestive history of heavy alcohol use, imaging tests to rule out obstructive etiologies should be pursued, but invasive tests including liver biopsy should not be pursued.
